# Greater adherence to the Healthy Nordic Food Index is associated with lower all-cause mortality in a population-based sample from northern Germany

**DOI:** 10.1007/s00394-023-03271-0

**Published:** 2023-10-19

**Authors:** Paula Stürmer, Ilka Ratjen, Katharina Susanne Weber, Cara Övermöhle, Tatjana Patricia Liedtke, Sabina Waniek, Eike Andreas Strathmann, Wolfgang Lieb

**Affiliations:** 1 Institute of Epidemiology, Kiel University, Niemannsweg 11, 24105 Kiel, Germany; 2 Department of Hematology and Oncology, University Hospital Schleswig Holstein, Kiel, Germany

**Keywords:** All-cause mortality, Cox regression, Healthy Nordic Food Index, Modified Mediterranean Diet Score, Dietary Approaches to Stop Hypertension score, General population

## Abstract

**Purpose:**

Dietary pattern scores reflecting a high intake of beneficial food groups were associated with reduced mortality risk. Data on associations of such dietary pattern scores in population-based samples from northern Germany are lacking. Therefore, we examined the association of three dietary pattern scores with all-cause mortality in a moderate-sized prospective sample from northern Germany.

**Methods:**

The study sample comprised 836 participants (43.8% females, median age 62.4 years). Based on a validated, self-administered Food Frequency Questionnaire, the dietary scores Dietary Approaches to Stop Hypertension (DASH), Modified Mediterranean Diet Score (MMDS), and Healthy Nordic Food Index (HNFI) were calculated. Cox proportional hazard regression models, adjusted for age, sex, body mass index, waist to hip ratio, education, smoking status, total energy intake, and physical activity, were used to separately relate DASH, MMDS, and HNFI to all-cause mortality.

**Results:**

During a median follow-up period of 11 years, 93 individuals died. While DASH and MMDS scores were not associated with all-cause mortality, greater adherence to HNFI was associated with lower mortality hazards (HR: 0.47 [95% CI 0.25–0.89] when comparing the highest score quartile to the lowest; HR: 0.79 [95% CI 0.64–0.98] for HNFI modeled as a 1-Standard Deviation increment). Among different HNFI components, higher intake of oats and cereals displayed the most conclusive association with all-cause mortality (HR: 0.59 [95% CI 0.38–0.91] when comparing high and low intake).

**Conclusion:**

In an elderly general population sample from northern Germany, we observed greater adherence to HNFI to be associated with lower all-cause mortality.

**Supplementary Information:**

The online version contains supplementary material available at 10.1007/s00394-023-03271-0.

## Introduction

Approximately, one in five deaths globally each year is associated with dietary risk factors, accounting for 11 million deaths worldwide in 2017. In the Global Burden of Disease Study, a diet too low in whole grains and fruits, as well as an excessive intake of sodium were identified as leading dietary risks [1]. On the other hand, an increased consumption of healthy foods and a decreased intake of detrimental foods are associated with a reduced mortality risk [2].

The Mediterranean Diet (MD) is a dietary pattern for which beneficial health effects have repeatedly been reported [3–5]. It is characterized by a high intake of healthy foods, such as fruits, vegetables, olive oil, and legumes, while limiting the consumption of foods like red meat and dairy products [6]. There is plenty of evidence that adherence to a MD is associated with a lower risk of all-cause mortality [7–13], yet this association seems to be more consistent and pronounced in Mediterranean populations than in study samples from non-Mediterranean regions [14–16]. A Healthy Nordic Diet, rich in health-promoting foods traditionally consumed in Nordic countries, is proposed as an alternative healthy dietary pattern in Western cultures outside the Mediterranean region [17, 18]. The Healthy Nordic Food Index (HNFI) is a dietary pattern score comprising of six healthy food groups (apples and pears, cabbage, root vegetables, fish, oatmeal, and wholegrain (rye) bread) [19]. Thus far, only a few prospective studies have investigated the association between this dietary pattern and mortality risk in population-based cohorts. In these analyses, greater adherence to the HNFI conferred lower mortality risks in a Danish cohort, in Swedish women, and in the European Investigation into Cancer and Nutrition (EPIC) cohort [19–22]. A third dietary pattern proposed as beneficial for human health is the Dietary Approaches to Stop Hypertension (DASH) diet. This diet is characterized by a high intake of fruits, vegetables, and low-fat dairy products, as well as a low consumption of sodium and saturated and total fat. Originally, this diet was developed as a non-pharmacological strategy to prevent or reduce hypertension [23]. Furthermore, increasing epidemiological evidence suggests that greater adherence to this dietary pattern is associated with a reduced mortality risk [7, 10, 13, 24].

In the present analysis, we aimed to assess the association of these three a priori dietary pattern scores—the Modified Mediterranean Diet Score (MMDS), HNFI, and DASH score—with all-cause mortality in an elderly sample from the North of Germany.

## Methods

### Study sample

The current analysis was conducted in a sub-sample of the control cohort of the popgen Biobank in Kiel, Germany, which is described in detail elsewhere [25]. In brief, between 2005 and 2007, 1316 individuals, recruited through population registries and from blood donors of the University Hospital in Kiel, were included in a reference sample, the “popgen controls”, initially used for genetic case–control analyses. The second examination cycle of this sample, conducted between 2010 and 2012, serves as the baseline examination for our analyses relating dietary patterns to mortality. At this second examination cycle, a physical examination by trained personnel was conducted and participants were asked to fill out standardized questionnaires on demographics and lifestyle factors, such as dietary habits and smoking status. All information regarding potential covariates were likewise obtained from the second examination cycle, thus from the baseline examination for our analyses. Vital status of participants was ascertained in 2022 via population registries. As no information about cause of death was provided, we only considered all-cause mortality for our analyses. After excluding individuals with missing physical examination data, or missing data on sex, education, and smoking status, as well as participants with implausible energy intake (women: < 500 or > 3500 kcal/d; men: < 800 or > 4000 kcal/d [26]) and those lost to follow-up, the analytical sample comprised of 836 individuals.

The study was approved by the ethical review board of the Medical Faculty of the Kiel University (project identification code A 156/03). All participants gave written informed consent.

### Medical examination

Anthropometric measurements were performed by trained personnel [27, 28]. Blood pressure was measured in duplicate (2-min interval between measurements) using a sphygmomanometer with the participants resting for at least 5 min before the first measurement. Hypertension was defined as systolic blood pressure  ≥ 140 mmHg, or diastolic blood pressure  ≥ 90 mmHG, or use of antihypertensive medication [29]. Diabetes was defined as glycated hemoglobin A1c  ≥ 6.5%, or fasting serum glucose  ≥ 126 mg/dL, or intake of glucose-lowering medication [30]. By use of a standardized self-administered questionnaire comprised of validated questions, participants reported the weekly hours spent in physical activity (walking, cycling, sports, gardening, housework, home repair, and stairs climbed per day) during the previous 12 months [31]. We calculated total physical activity by summing up the Metabolic Equivalent of Task (MET) values assigned to each activity [32, 33].

### Dietary assessment

Dietary intake over the previous 12 months was assessed at the second examination cycle of the “popgen controls” using a validated, self-administered, semi-quantitative Food Frequency Questionnaire (FFQ), initially designed for the German Potsdam sample of the EPIC project [34]. Participants were asked to report the frequency of consumption of 112 foods and beverages during the previous year. Micro- and macronutrient content as well as food group and energy intake were then estimated using the German Food Code and Nutrient Database (version II.3) [35]. In our analyses, we considered the following a priori dietary scores:

#### Dietary Approaches to Stop Hypertension score

The DASH score was calculated according to Fung et al. [36] based on sex-specific quintiles of intake of defined food groups. Consumption of fruits, vegetables, nuts and legumes, low-fat dairy products, and whole grains was scored positively with the highest quintile receiving 5 points and the lowest quintile receiving 1 point. Foods presumed to be detrimental (dietary sodium, red and processed meat, and sweetened beverages) were scored reversely. Added up, the DASH score theoretically ranged from 8 to 40 points.

#### Modified Mediterranean Diet Score

For the MD, we calculated the MMDS according to Trichopoulou et al. [37]. Values of 0 and 1 were assigned to consumption of nine food groups using sex-specific medians of the study sample as cut-points. For food groups considered beneficial (vegetables, legumes, fruits and nuts, fish and seafood, and cereals), 1 point was assigned to consumption at or above the median and 0 points to intake below median. Consumption of food groups presumed to be detrimental (meat and poultry and dairy products) were scored reversely. As a dietary component considered beneficial in moderate consumption, alcohol intake was scored as a dichotomous variable with 1 point assigned to an intake of 5–25 g/d for women and 10–50 g/d for men, respectively. Otherwise, a value of 0 was assigned. As in non-Mediterranean countries polyunsaturated fatty acids constitute the principal unsaturated fats in diet, the ratio of the sum of monounsaturated and polyunsaturated fatty acids to saturated fatty acids was applied rather than the monounsaturated:saturated lipids ratio originally introduced by Trichopoulou et al. [8, 9]. 1 point was assigned to subjects with intake at or above median and 0 points for consumption below median. Summed up, the MMDS showed a theoretical range from 0 to 9 points.

#### Healthy Nordic Food Index

The HNFI comprises of six food items commonly consumed in Nordic countries and was originally developed by Olsen et al. [19]. Consumption of fish, root vegetables, cabbage, and apples and pears was scored according to sex-specific medians of the study sample with intake above the median scoring 1 point and intake below the median scoring 0 points. Since the FFQ used in this study did not include an individual question about rye bread, one minor adjustment was undertaken by considering wholegrain bread instead of rye bread [20]. The same applies for the variable oatmeal. Therefore, we considered total breakfast cereals and termed this variable “oats and cereals”. As intake of wholegrain bread and oats and cereals was relatively low in our sample, instead of using pre-defined cut-off values for these food groups as defined by Olsen et al. (rye bread  ≥ 63 g/d, oatmeal  ≥ 21 g/d [19]), the scoring method using sex-specific sample medians was applied for these food groups as well [38]. Added up, HNFI ranges from 0 to 6 points.

### Statistical analysis

Categorical variables were reported as absolute numbers and percentages and continuous variables as median and interquartile range. Prior to all analyses, we performed an adjustment for total energy intake for the dietary scores and defined food groups, respectively. Here, we used the residual method with subsequently adding a constant based on mean energy intake (2197.48 kcal/d) of the study sample [39]. For two-sided tests, statistical significance was considered at *p*  ≤  0.05. We performed all analyses with SAS Enterprise Guide 7.1 (SAS Institute, Cary, NC, USA).

#### Association of adherence to defined dietary patterns with mortality

We categorized participants into quartiles of dietary scores, with a higher quartile indicating higher adherence to the respective dietary pattern. Quartile 1 was set as the reference. First, the unadjusted association of dietary scores with survival was graphically displayed using Kaplan–Meier curves, showing the survival time for each dietary score quartile. Cox proportional hazard regression models were used to adjust for relevant confounders. Model 1 adjusted for sex and age. Model 2 additionally included body mass index (BMI; kg/m^2^), waist to hip ratio, years of education (≤ 9, 10,  ≥ 11 years), smoking status (never [≤ 3 months duration], former [≥ 3 months duration], and current smokers), physical activity (MET-h/week), and energy intake (kcal/d). As MMDS already considers alcohol intake, we tested if alcohol intake (g/d) was a confounder when analyzing DASH and HNFI in the Cox regression model. The addition of alcohol intake did not affect the model, and was, therefore, not included in the final analysis. Time to event was modeled for each dietary score quartile, using the bottom quartile as the reference, with all-cause mortality as the endpoint. When modeled on a continuous scale, effect estimates were provided per 1-Standard Deviation (SD) increments for each score to enhance comparability. Hazards ratios (HRs) and 95% confidence intervals (95% CI) were used to quantify these associations. The underlying time variable was the time alive (in years) between dietary assessment and death or censoring in 2022. To assess the validity of the proportional hazard assumption, we used the Schoenfeld residuals method and inclusion of time interaction terms in the model. Age did not meet the proportional hazard assumption and a corresponding time interaction term (survival time × age) was, therefore, added to the Cox regression models.

#### Stratified analyses, assessment of interactions, and sensitivity analysis

After analyses in the overall sample, we performed multivariable-adjusted survival analyses stratified by sex, BMI classes (≤ 24.9 kg/m^2^, 25.0–29.9 kg/m^2^,  ≥ 30.0 kg/m^2^), smoking habits, and diabetes status (present vs. absent) (covariates ascertained at time of dietary assessment). To test for effect modification, we included multiplicative interaction terms of dietary scores with the respective variables in the fully adjusted Cox regression model. To rule out possible reverse causation, we performed a sensitivity analysis excluding participants who died within 1 year after dietary assessment.

#### Association of adherence to defined food groups with mortality

For dietary scores significantly associated with the risk of all-cause mortality, we performed additional multivariable-adjusted analyses to understand which food groups within the score drive its associations with mortality. First, we used intake of defined food groups (low vs. high intake, based on intake < sample median and intake ≥ sample median) as the exposure and all-cause mortality as the endpoint. Second, we omitted individual food groups from the scores and assessed how their association with all-cause mortality risk changed when food groups were selectively excluded.

## Results

### Characterization of the study sample

Analyses were performed in an elderly and slightly overweight study sample (median age 62.4 years, median BMI 26.7 kg/m^2^ at dietary assessment, respectively) with a female share of 43.8%. Table 1 displays the general characteristics of the study sample. Participants of the “popgen controls” that were excluded from the analysis were younger as well as more likely to be current smokers and to have a higher school education than those included in the study sample (Online Resource 1). Food items that contribute to food groups used to calculate dietary pattern scores can be found in Online Resource 2. Energy-adjusted dietary scores showed an interquartile range from 20.4 to 27.0 for DASH, from 3.9 to 6.0 for MMDS, and from 2.0 to 4.2 for HNFI. Out of 836 subjects at time of dietary assessment, 93 died during a median follow-up period of 11 years. Individuals who died during this period were more likely to be male and were older at time of dietary assessment. Furthermore, they were more likely to be former smokers and had a lower level of education, showed a higher prevalence of diabetes, and scored slightly lower on HNFI as compared to individuals who were alive at the end of the follow-up period. Participants´ median daily intakes of energy-adjusted food groups used for calculation of dietary scores are presented in Online Resource 3.

**Table 1:** Characterization of the overall study sample and stratified by mortality status

	Total sample (*n* = 836)	Deceased^1^ (*n* = 93)	Alive^1^ (*n* = 743)
Survival time in years^1^	11 [10; 11]	8 [5; 10]	11 [10; 12]
Female sex, *n* (%)	366 (43.8%)	27 (29.0%)	339 (45.6%)
Age in years	62.4 [55.1; 71.1]	71.9 [64.1; 76.5]	61.7 [54.1; 70.1]
Height in cm	171.5 [164.0; 178.5]	170.0 [164.0; 176.5]	172.0 [164.0; 178.5]
Weight in kg	79.5 [69.6; 90.7]	76.2 [67.5; 87.7]	79.7 [69.8; 91.0]
BMI in kg/m^2^	26.7 [24.3; 29.7]	26.6 [24.2; 30.1]	26.7 [24.3; 29.6]
WHR	0.94 [0.88; 1.00]	0.98 [0.92; 1.03]	0.94 [0.88; 1.00]
Diabetes, *n* (%)	78 (9.3%)	20 (21.5%)	58 (7.8%)
Hypertension^3^, *n* (%)	537 (64.3%)	73 (78.5%)	464 (62.5%)
Physical Activity in MET-h/week	88.6 [57.5; 129.2]	77.0 [53.0; 123.6]	90.0 [58.5; 130.5]
DASH	24 [20; 27]	24 [22; 27]	24 [20; 27]
DASH (energy-adjusted)^2^	23.7 [20.4; 27.0]	24.4 [22.0; 27.0]	23.6 [20.3; 27.1]
MMDS	5 [4; 6]	5 [4; 6]	5 [4; 6]
MMDS (energy-adjusted)^2^	5.0 [3.9; 6.0]	4.7 [3.5; 5.7]	5.0 [3.9; 6.0]
HNFI	3 [2; 4]	3 [1; 4]	3 [2; 4]
HNFI (energy-adjusted)^2^	3.1 [2.0; 4.2]	2.9 [1.6; 4.2]	3.1 [2.0; 4.2]
Energy intake in kcal/d	2106.7 [1753.6; 2564.2]	2165.2 [1823.3; 2605.3]	2100.6 [1746.7; 2555.7]
Men	2367.4 [2004.2; 2798.9]	2315.0 [1934.4; 2723.9]	2378.7 [2010.2; 2800.7]
Women	1821.6 [1590.9; 2129.5]	1650.5 [1441.6; 2206.1]	1828.0 [1597.2; 2115.1]
Education			
≤ 9 years, *n* (%)	287 (34.3%)	44 (47.3%)	243 (32.7%)
10 years, *n* (%)	281 (33.6%)	23 (24.7%)	258 (34.7%)
≥ 11 years, *n* (%)	268 (32.1%)	26 (28.0%)	242 (32.6%)
Smoking status			
Never, *n* (%)	353 (42.2%)	28 (30.1%)	325 (43.7%)
Current, *n* (%)	109 (13.0%)	10 (10.8%)	99 (13.3%)
Former, *n* (%)	374 (44.7%)	55 (59.1%)	319 (42.9%)

*BMI* body mass index, *cm* centimeters, *d* day, *DASH* Dietary Approaches to Stop Hypertension, *HNFI* Healthy Nordic Food Index, *kcal* kilocalories, *kg* kilograms, *m* meters, *MET-h* Metabolic Equivalent of Task in hours, *MMDS* Modified Mediterranean Diet Score, *WHR* waist to hip ratio

^1^At mortality follow-up in 2022

^2^Dietary scores adjusted for total energy intake using residual method and subsequent addition of a constant based on mean energy intake of the study population

^3^Because of missing values of blood pressure in one subject, here total sample *n* = 835

Values of categorical variables are *n* (%) and of continuous variables are median [IQR]. All values except for survival time and mortality status are baseline characteristics collected at the second examination cycle of the popgen control cohort

### Association of defined dietary pattern scores with survival

**Fig. 1:**
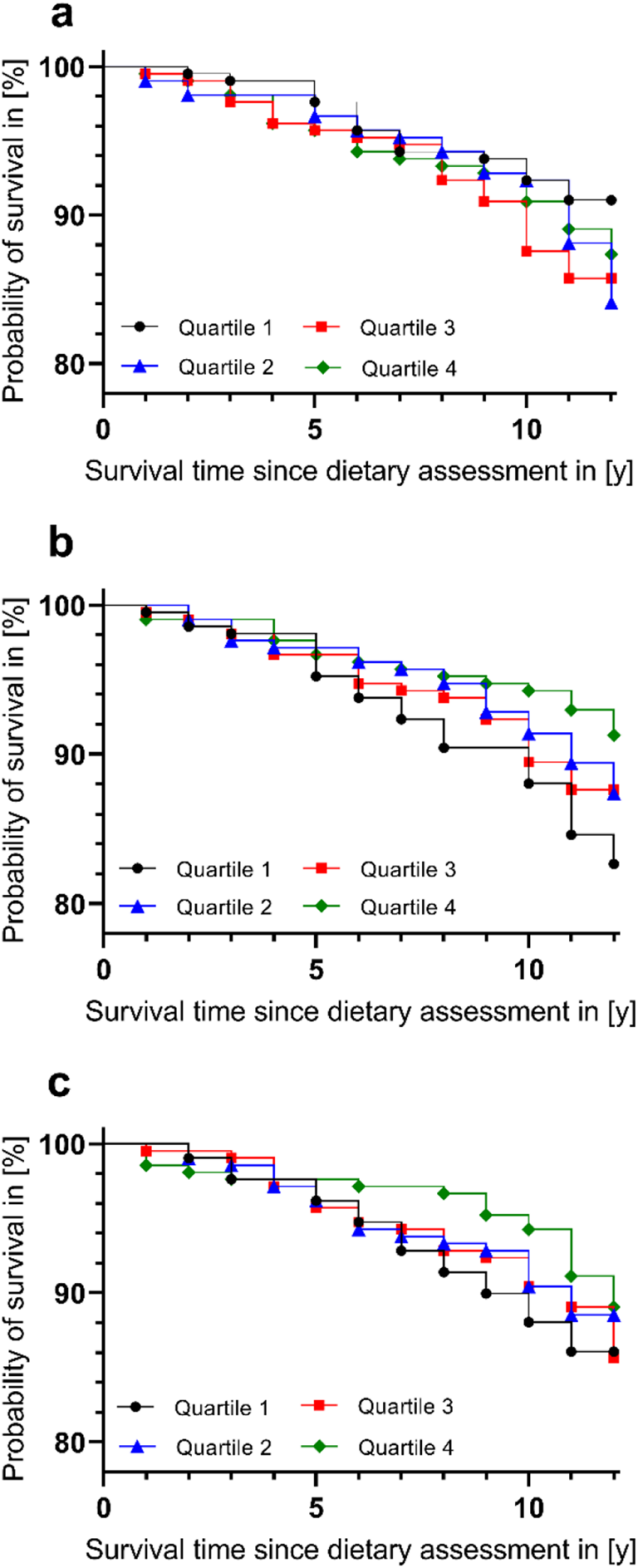
Kaplan–Meier curves displaying the association of energy-adjusted dietary scores (**a** DASH, **b** MMDS, **c** HNFI) with all-cause mortality. By use of Log-Rank test, no association between dietary scores and all-cause mortality was found (DASH: *p* = 0.4606, MMDS: *p* = 0.4564, HNFI: *p* = 0.0894). We used dietary scores adjusted for total energy intake using residual method and subsequent addition of a constant based on mean energy intake of the study sample. Artwork was created using GraphPad Prism version 9.4.1, GraphPad Software. *DASH* Dietary Approaches to Stop Hypertension, *HNFI* Healthy Nordic Food Index, *MMDS* Modified Mediterranean Diet Score, *y* years

Figure 1 displays Kaplan–Meier curves for energy-adjusted dietary scores. Log-rank tests indicate no significant association with all-cause mortality for either score considered (DASH: *p* = 0.4606, MMDS: *p* = 0.4564, HNFI: *p* = 0.0894). Using multivariable-adjusted Cox proportional hazard regression models, DASH and MMDS scores were not associated with all-cause mortality (Table 2). However, for HNFI, participants in the fourth quartile (indicating greater adherence to the score) had significantly lower hazards of dying than individuals in the first quartile (HR: 0.44 [95% CI 0.24–0.82] after adjustment for sex and age). This association persisted upon multivariable adjustment (HR: 0.47 [95% CI 0.25–0.89]). When modeled as a continuous trait, a 1-SD increment of energy-adjusted HNFI was associated with a 24% decline in mortality hazards after adjustment for sex and age ([95% CI 0.62–0.94]) and with a 21% decline after multivariable adjustment ([95% CI 0.64–0.98]). In a sensitivity analysis, after excluding participants who died within 1 year after dietary assessment, results did not change substantially (data not shown).

**Table 2:** Association of energy-adjusted dietary scores DASH, MMDS, and HNFI (modeled in quartiles and as continuous traits) with all-cause mortality using Cox proportional hazard regression models

	1-SD increment^2^	Quartiles^1^			
		1^3^	2	3	4
DASH					
All individuals, *n* (%)	836 (100.0%)	209 (25.0%)	209 (25.0%)	209 (25.0%)	209 (25.0%)
Deceased, *n* (%)	93 (11.1%)	17 (8.1%)	26 (12.4%)	28 (13.4%)	22 (10.5%)
Score	24 [20; 27]	18 [17; 20]	22 [21; 23]	25 [24; 26]	30 [29; 31]
Score (energy-adjusted)^4^	23.7 [20.4; 27.0]	18.4 [16.6; 19.4]	22.4 [21.4; 23.0]	25.2 [24.6; 26.2]	29.5 [28.2; 30.8]
Model 1	0.91 [0.74–1.13]	Ref	0.97 [0.52–1.81]	1.07 [0.59–1.95]	0.87 [0.47–1.63]
Model 2	0.95 [0.77–1.19]	Ref	1.05 [0.56–1.96]	1.12 [0.66–2.19]	1.00 [0.53–1.90]
MMDS					
All individuals, *n* (%)	836 (100.0%)	209 (25.0%)	209 (25.0%)	209 (25.0%)	209 (25.0%)
Deceased, *n* (%)	93 (11.1%)	28 (13.4%)	23 (11.0%)	24 (11.5%)	18 (8.6%)
Score	5 [4; 6]	3 [2; 3]	4 [4; 4]	5 [5; 6]	7 [7; 8]
Score (energy-adjusted)^4^	5.0 [3.9; 6.0]	3.0 [2.2; 3.3]	4.2 [4.1; 4.7]	5.2 [5.1; 5.8]	6.9 [6.2; 7.2]
Model 1	0.87 [0.71–1.06]	Ref	0.75 [0.43–1.31]	0.91 [0.53–1.57]	0.65 [0.36–1.18]
Model 2	0.88 [0.72–1.07]	Ref	0.76 [0.44–1.33]	0.98 [0.56–1.71]	0.67 [0.37–1.23]
HNFI					
All individuals, *n* (%)	836 (100.0%)	209 (25.0%)	209 (25.0%)	209 (25.0%)	209 (25.0%)
Deceased, *n* (%)	93 (11.1%)	31 (14.8%)	22 (10.5%)	25 (12.0%)	15 (7.2%)
Score	3 [2; 4]	1 [0; 1]	3 [2; 3]	4 [4; 4]	5 [5; 6]
Score (energy-adjusted)^4^	3.1 [2.0; 4.2]	1.2 [0.7; 1.5]	2.4 [2.2; 2.8]	3.5 [3.3; 3.8]	4.8 [4.4; 5.3]
Model 1	**0.76 [0.62**–**0.94]**	Ref	**0.56 [0.33**–**0.98]**	0.68 [0.40–1.15]	**0.44 [0.24**–**0.82]**
Model 2	**0.79 [0.64**–**0.98]**	Ref	**0.56 [0.32**–**0.98]**	0.77 [0.45–1.32]	**0.47 [0.25**–**0.89]**

Scores are in median [interquartile range] and associations in hazard ratios [95% confidence interval] with bold figures indicating significant associations (*p* < 0.05)

Model 1: adjusted for sex and age

Model 2: Model 1 further adjusted for body mass index, waist to hip ratio, education, smoking status, total energy intake, and physical activity

*DASH* Dietary Approaches to Stop Hypertension, *HNFI* Healthy Nordic Food Index, *MMDS* Modified Mediterranean Diet Score, *Q* quartile, *SD* standard deviation

^1^Cut-off values for energy-adjusted dietary pattern scores are as follows: DASH, Q1: 9.91–20.43; Q2: 20.45–23.70; Q3: 23.70–27.01; Q4: 27.05–35.94; MMDS: Q1: 0.80–3.91; Q2: 3.91–4.98; Q3: 4.98–6.00; Q4: 6.00–9.11; HNFI: Q1: -0.74–1.95; Q2: 1.96–3.07; Q3: 3.08–4.17; Q4: 4.18–6.54

^2^To increase comparability between dietary scores, 1-SD increments of dietary scores were used

^3^Quartile 1 was set as reference

^4^For survival analyses, we used dietary scores adjusted for total energy intake using residual method and subsequent addition of a constant based on mean energy intake of the study sample

### Assessment of interactions and stratified analyses

We tested for associations between the energy-adjusted dietary scores (HR per 1-SD increments) and mortality risk stratified by potential effect modifiers in the fully adjusted Cox regression model (Table 3). Individuals with diabetes seemed to benefit more strongly from greater adherence to MMDS or HNFI as compared to subjects without the condition, resulting in a lower HR for mortality among those with diabetes (HR: 0.51 [95% CI 0.30–0.88] for MMDS; HR: 0.39 [95% CI 0.21–0.73] for HNFI; considered as 1-SD increments, respectively) and no association among individuals without diabetes (*p* = 0.2382 for interaction between MMDS and diabetes, *p* = 0.0251 for interaction between HNFI and diabetes,). Although interaction terms failed to reach statistical significance for smoking and BMI categories, we observed slight differences in the association pattern for distinct subgroups. For current smokers, an association between greater adherence to DASH and HNFI and improved survival could be shown (HR: 0.40 [95% CI 0.17–0.94] for DASH; HR: 0.41 [95% CI 0.18–0.94] for HNFI), whereas these dietary scores were not associated with survival in never and former smokers. Furthermore, we observed a protective effect of greater adherence to HNFI in individuals with obesity (BMI ≥ 30.0 kg/m^2^; HR: 0.64 [95% CI 0.41–0.99]), but no statistically significant associations between dietary scores and survival in the other BMI strata. Considering sex, no statistical interaction was observed.

**Table 3:** Association of energy-adjusted dietary scores DASH, MMDS, and HNFI with all-cause mortality using Cox proportional hazard regression models stratified by smoking status, BMI classes, and pre-existing diabetes, respectively

	Smoking status				BMI classes				Diabetes		
	Never (*n* = 353)	Current (*n* = 109)	Former (*n* = 374)	*p* value^3^	≤ 24.9 kg/m^2^ (*n* = 257)	25.0–29.9 kg/m^2^ (n = 387)	≥ 30.0 kg/m^2^ (*n* = 192)	*p* value^3^	No (*n* = 758)	Yes (*n* = 78)	*p* value^3^
DASH^1^ (energy-adjusted)^2^	1.34 [0.85–2.11]	**0.40 [0.17**–**0.94]**	0.96 [0.73–1.27]	0.4690	0.70 [0.48–1.01]	1.25 [0.88–1.78]	1.26 [0.78–2.04]	0.0842	0.99 [0.77–1.28]	0.85 [0.47–1.52]	0.5825
MMDS^1^ (energy-adjusted)^2^	0.80 [0.54–1.18]	0.51 [0.21–1.21]	0.97 [0.74–1.26]	0.3162	0.81 [0.55–1.21]	0.99 [0.73–1.35]	0.83 [0.55–1.25]	0.9925	0.92 [0.73–1.16]	**0.51 [0.30**–**0.88]**	0.2382
HNFI^1^ (energy-adjusted)^2^	0.86 [0.57–1.30]	**0.41 [0.18**–**0.94]**	0.85 [0.65–1.11]	0.7078	0.76 [0.53–1.09]	1.00 [0.70–1.43]	**0.64 [0.41**–**0.99]**	0.7808	0.87 [0.69–1.11]	**0.39 [0.21**–**0.73]**	**0.0251**

*BMI* body mass index, *DASH* Dietary Approaches to Stop Hypertension, *HNFI* Healthy Nordic Food Index, *kg* kilograms, *m* meters, *MMDS* Modified Mediterranean Diet Score

^1^To increase comparability between dietary scores, 1-Standard Deviation increments were used as continuous scores

^2^For stratified survival analyses, we used dietary scores adjusted for total energy intake using residual method and subsequent addition of a constant based on mean energy intake of the study sample

^3^p values indicate interaction between variables and dietary scores

Values are in hazard ratios [95% confidence interval] with bold figures indicating statistical significance (*p* < 0.05). Analyses were adjusted for sex, age, BMI, waist to hip ratio, education, smoking status, total energy intake, and physical activity

### Association of defined food groups of the HNFI with survival

To elucidate which food groups are mainly responsible for the association of HNFI with survival, we calculated multivariable-adjusted Cox proportional hazards for the different components of the HNFI (cabbage, root vegetables, apples and pears, fish, oats and cereals, and wholegrain bread), comparing low vs. high intake for each of these food groups. Consumption of oats and cereals was inversely related to all-cause mortality (HR: 0.59 [95% CI 0.38–0.91]; Online Resource 4). Furthermore, reduced mortality hazards with higher adherence to HNFI did not persist upon exclusion of either root vegetables, fish, or oats and cereals intake from the full score (HR: 0.88 [95% CI 0.71–1.10]; HR: 0.85 [95% CI 0.69–1.06]; HR: 0.86 [95% CI. 0.69–1.07], respectively; Online Resource 5).

## Discussion

In a moderate-sized elderly sample from northern Germany, we assessed the associations between three a priori dietary scores and all-cause mortality over an 11-year follow-up period (93 deaths). While we did not detect overall associations between DASH score and MMDS and all-cause mortality, a 1-SD increment in adherence to HNFI was associated with a 21% reduction in hazard of all-cause mortality after multivariable adjustment.

### Association of HNFI with lower all-cause mortality

Our results are in line with previous studies reporting inverse associations of adherence to HNFI with all-cause mortality in three population-based Scandinavian cohorts [19–21] and in a large sample of the EPIC cohort [22]. A recent meta-analysis reported that the Healthy Nordic Diet positively affects various cardiovascular risk factors, including lipid levels, and also reduces the risk for the development of various cardiometabolic outcomes, such as diabetes and clinically overt cardiovascular disease [40]. These associations are an important explanation for the observed association of the HNFI with all-cause mortality. Of the six food groups comprising the HNFI, we identified oats and cereals intake to be the potential driver of this association as a higher consumption of oats and cereals was independently associated with lower all-cause mortality. When we excluded oats and cereals from HNFI, the association between all-cause mortality and adherence to this modified dietary score failed to show statistical significance, further emphasizing the relevance of oats and cereals as a HNFI score component.

### Lack of association of DASH score and MMDS with all-cause mortality

As opposed to HNFI, in our sample from northern Germany, the MMDS was not associated with all-cause mortality. This is in contrast to previously reported analyses in Western cohorts that showed reduced mortality risks with greater adherence to a MD score [7–13, 21, 22, 37, 41–44]. At that, reduced mortality hazards were even more pronounced in Mediterranean populations [16], whereas results from studies in Western non-Mediterranean regions sometimes yield conflicting results [14, 15]. To the best of our knowledge, this is the first study analyzing adherence to a MD score in relation to all-cause mortality risk in a population-based sample from Germany outside the German EPIC cohort [22, 37]. Interestingly, when only considering the German sub-sample of the EPIC cohort, Trichopoulou et al. failed to show an inverse association of adherence to MMDS and all-cause mortality; while in the total EPIC sample, greater adherence to this score was associated with reduced mortality risks [37].

The various published MD scores differ in scoring methods used, energy-adjustments applied, and food groups considered [10, 16, 45] which hinders comparisons between different studies. When comparing the associations between four differently generated MD scores and mortality risk in a UK population-based sample, results from the MD score based on Trichopoulou et al. [8] differed from other scores considered [11]. This score—as well as its modified version [37] applied in our study—uses sex-specific sample medians of food group consumption to classify the intake of individuals as “more adherent” vs. “less adherent” to the MD. Importantly, this scoring method does not consider the absolute intake of these food groups. Thus, individuals considered as adherent to the MD based on this relative classification scheme might still have rather low absolute intakes of typical Mediterranean foods.

Energy-adjusted consumption of various food groups differ considerably between our study and some studies in which an inverse association between adherence to MD scores and all-cause mortality was found [9, 43, 44]. In comparison, consumption of beneficial score components like vegetables, fruits, and legumes was considerably lower in our study sample. As intake of these food groups was independently associated with lower mortality risks in a meta-analysis [2], their relatively low consumption in our sample—which is, on average, also considerably lower than the German recommendations [46]—might have impeded an association between adherence to MMDS and mortality risk.

In addition, no association of DASH score with all-cause mortality was observed in our sample, which is in contrast to previously reported analyses in population-based studies [7, 10, 13, 24, 41]. Like the MMDS, the DASH score is based on the sample-specific relative intake of food groups instead of considering food group intake in absolute terms. Amongst others, the DASH score also considers vegetable and fruit intake, which was shown to be considerably lower in our study sample compared to studies showing improved survival with greater adherence to MD scores [9, 43, 44].

Scoring high on dietary scores based on sample-specific relative intakes of food groups does not necessarily implicate high absolute consumption of food groups and therefore a true strong adherence to the underlying dietary pattern [11, 16]. The lack of association of adherence to DASH score and MMDS and all-cause mortality risk in our sample might, therefore, be explained by the important principle of generating the dietary scores using sample-specific relative intake of food groups instead of considering absolute consumption.

### Dietary score adherence and survival, stratified by prevalence of diabetes, smoking, and BMI categories

In analyses stratified by defined risk factors, improved survival with greater adherence to HNFI was particularly pronounced in individuals with diabetes and failed to reach statistical significance in those without the condition. As one of the characteristics of diabetes, hyperglycemia is associated with a higher risk for adverse health outcomes, including all-cause mortality [47]; whereas, improved glycemic control by certain pharmacological regimens was associated with reduced risks for all-cause mortality in individuals with type 2 diabetes [48]. β-glucan, a soluble dietary fiber in oat, is known to lower postprandial glycemic responses [49]. Thus, individuals with diabetes might benefit from postprandial glucose-lowering effects of β-glucan [50] by intake of oats, which partly comprise the food group oats and cereals. Even though oats and cereals intake and, therefore, also β-glucan intake is very low in our cohort, this adds to the possible explanation how adherence to HNFI might be particularly beneficial in this sub-sample.

Furthermore, in contrast to results in the overall study sample, in individuals with diabetes also greater adherence to MMDS was inversely associated with lower all-cause mortality risk. The MMDS does not consider oats and cereals as a score component but includes cereals, a heterogeneous food group comprising different wholegrain and refined grain products as well as oats and cereals. Still, the effect on blood-glucose control by β-glucan in oats included in the food group cereals might account for the beneficial impact on mortality risk in individuals with diabetes and greater adherence to MMDS, even though the very low intake of oats and cereals in our study sample needs to be considered in this context.

Apart from this, to the best of our knowledge, we are the first to report greater adherence to DASH score and HNFI to be significantly associated with a reduction in all-cause mortality risk in current smokers. Amongst others, smoking contributes to enhanced oxidative stress which increases the risk for adverse health outcomes [51]. Beneficial food groups, like fruits and vegetables, are rich in bioactive compounds with antioxidant capacities that counteract oxidative stress responses [52]. High HNFI or DASH scores implicate a higher relative intake of such beneficial food groups. This is one of various biological mechanisms possibly explaining an especially protective effect of such a healthful diet in current smokers. The same might apply for individuals with obesity as excessive body fat also contributes to inflammation caused by oxidative stress [53]. When stratifying by BMI, we found individuals with obesity to benefit from adherence to HNFI in terms of improved survival. However, as no data on antioxidative capacity of specific foods were available in our cohort, this point is speculative and requires further investigation.

Overall, our findings indicate that adherence to health-promoting dietary habits, represented by scoring higher on DASH score, MMDS, or HNFI, might be even more beneficial for individuals at risk, than for people without such health risk factors. However, as only the interaction between diabetes and HNFI adherence showed statistical significance, interpretation of these findings warrant caution and require further investigation in future studies.

### Strengths and limitations

Strengths of this study include its prospective and population-based design, the comprehensive assessment of diet and potential covariates using established instruments and methods, as well as the small number of participants lost to follow-up.

Still, some limitations merit consideration. Even though the FFQ used in this study is a well-established dietary assessment tool, its questions are not perfectly designed to assess the intake of food groups required for the different scores applied. For example, as the intake of oatmeal is not specifically asked in the FFQ, we had to use the food group oats and cereals that results from a question that also comprises other breakfast cereals besides oats. Similar limitations also apply for the MMDS and the DASH score. As we only considered data about dietary intake from the second examination cycle of the “popgen controls”, potential changes in dietary habits over time influencing mortality risk could not be taken into account. Furthermore, a certain heterogeneity considering healthy lifestyle factors was observed in the study sample when comparing individuals scoring highest on any of the three dietary scores with those scoring lowest. In quartile 4, individuals were more likely to be physically active, less likely to be current smokers, and tended to have a lower BMI (data not shown). Even though we considered such covariates in our multivariable-adjusted model, we cannot entirely rule out residual confounding by other factors correlating with a healthy lifestyle. From our original sample, we had to exclude a total of 99 individuals due to missing data. The excluded individuals were slightly younger and more likely to be current smokers as compared to the analytical sample. Finally, since our sample comprises elderly adults from a specific German region, the generalizability of our results is limited.

## Conclusion

In conclusion, we related adherence to three established dietary pattern scores, namely DASH score, MMDS, and HNFI, with all-cause mortality in an elderly population-based sample from northern Germany. We observed greater adherence to HNFI to be associated with longer survival, with oats and cereals consumption as a potential driver of this association. In analyses stratified by risk factors, this association was most prominent in individuals with diabetes. Our results indicate that scoring high on the HNFI might be more predictive of reduced mortality risks in a northern German population than adherence to the MMDS or DASH score. This might be due to northern German eating habits showing higher resemblance to the Healthy Nordic Diet than to a traditional MD or DASH diet, possibly because of its geographical proximity to Scandinavian countries, where the HNFI was first developed and applied [19]. Recommendations for a healthy dietary lifestyle in northern Germany should, therefore, focus on promoting adherence to a Healthy Nordic Diet fitting the geographical setting of this region.

### Supplementary Information

Below is the link to the electronic supplementary material.

Supplementary file1 (PDF 200 KB)

## Data Availability

Data described in the article, code book, and analytic code will be made available upon reasonable request through our P2N data application platform (https://portal.popgen.de/).
