# Supplementary material for: Greater adherence to the Healthy Nordic Food Index is associated with lower all-cause mortality in a population-based sample from northern Germany
Source: Eur J Nutr. 2023 Oct 19;63(2):365–75. doi: 10.1007/s00394-023-03271-0 (PMC10899306; doi:10.1007/s00394-023-03271-0)
Supplement: Supplementary file 1 — Supplementary file1 (PDF 200 KB) [file 394_2023_3271_MOESM1_ESM.pdf]

Greater adherence to the Healthy Nordic Food Index is associated with lower all-cause mortality in a population-based sample from northern Germany

European Journal of Nutrition

Paula Stürmer, Ilka Ratjen, Katharina Susanne Weber, Cara Övermöhle, Tatjana Patricia Liedtke, Sabina Waniek, Eike Andreas Strathmann, Wolfgang Lieb

Corresponding author: Paula Stürmer, Institute of Epidemiology, Kiel University, Niemannsweg 11, 24105 Kiel, Germany, paula.stuermer@epi.uni-kiel.de

Online Resource 1: Characterisation of participants of the "popgen controls" included vs. excluded from the analysis associating dietary patterns with all-cause mortality.

	**Included (n=863)**	**Excluded[1] (n=99)**	***p value***
**Female sex[2], n (%)**	366 (43.8%)	35 (36.5%)	0.1700
**Age[3] in years**	62.4 [55.1; 71.1]	51.1 [37.0; 64.5]	**<.0001**
**BMI[4] in kg/m²**	26.7 [24.3; 29.7]	27.3 [24.7; 30.1]	0.3064
**Energy intake in kcal/d**	2106.7 [1753.6; 2564.2]	2605.6 [1977.2; 4043.6]	**<.0001**
**Alcohol intake in g/d**	8.7 [3.2; 18.2]	10.6 [3.2; 23.4]	0.1473
**Smoking[5]**			**0.0130**
**Never, n (%)**	353 (42.2%)	36 (39.6%)	
**Current, n (%)**	109 (13.0%)	22 (24.2%)	
**Former, n (%)**	374 (44.7%)	33 (36.3%)	
**Education[6]**			**0.0465**
**<=9 years, n (%)**	287 (34.3%)	34 (35.8%)	
**10 years, n (%)**	281 (33.6%)	21 (22.1%)	
**>=11 years, n (%)**	268 (32.1%)	40 (42.1%)	

[1] Excluded from final study population due to missing physical examination data, or missing data on sex, education, and smoking status, as well as due to implausible energy intake (women: <500 or >3500 kcal/d; men: <800 or >4000 kcal/d (Willet 2013)) or loss to follow-up.
[2] In group of excluded participants due to missing data, here n=96.
[3] In group of excluded participants missing information on age was calculated based on data from self-administered questionnaires, as participants did not take part in the physical examination.
[4] In group of excluded participants due to missing data (participants did not take part in the physical examination), here n=88.
[5] In group of excluded participants due to missing data, here n=91.
[6] In group of excluded participants due to missing data, here n=95.

Online Resource 2: Food items contributing to food groups used to calculate dietary pattern scores.

**Dietary Approaches to Stop Hypertension score**	
Fruits[1]	Apple, pear, orange, mandarin, kiwi, pineapple, banana, fig, physalis, pomegranate, cherry, plum, apricot, mirabelle, nectarine, peach, berries, melon, grape, mixed fruits, dried fruits, olives
Vegetables[1]	Carrot, kohlrabi, tomato, pepper, cucumber, gherkin, avocado, green salad, onion, garlic, sauerkraut, spinach, broccoli, cauliflower, white and red cabbage, kale, brussel sprouts, asparagus, aubergine, courgette, mixed vegetables
Nuts and legumes[1]	Beans, chickpeas, peas, lentils, nuts, seeds, tofu
Low-fat dairy products[1]	Milk, dairy drink (fat content: 0.3% and 1.5%, respectively), (fruit) yogurt, buttermilk (fat content: 0.1 %, 0.3% and 1.5%, respectively), low-fat soft and hard cheese
Whole grains[1]	Bread and bread rolls (grey, rey, brown, wholegrain), crispbread
Dietary sodium[2]	Sodium
Red and processed meat[2]	Pork, beef, lamb, game, offal
Sweetened beverages[2]	Lemonade, soft drinks
**Modified Mediterranean Diet Score**	
Fruits and nuts[1]	Apple, pear, orange, mandarin, kiwi, pineapple, banana, fig, physalis, pomegranate, cherry, plum, apricot, mirabelle, nectarine, peach, berries, melon, grape, mixed fruits, dried fruits, nuts, seeds
Vegetables[1]	Carrot, kohlrabi, tomato, pepper, cucumber, gherkin, avocado, green salad, onion, garlic, sauerkraut, spinach, broccoli, cauliflower, white and red cabbage, kale, brussel sprouts, asparagus, aubergine, courgette, mixed vegetables
Legumes[1]	Beans, chickpeas, peas, lentils
Fish and seafood[1]	Fish, ready-to-eat fish dishes, mussels, crustaceans
Cereals[1]	Bread, bread roll, crispbread, potatoes, potato products, rice, pasta, grains, breakfast cereals, doughs, savoury biscuits
Meat and poultry[2]	Pork, beef, poultry, lamb, calf, rabbit, game, sausages, offal
Dairy products[2]	Milk, dairy drinks, buttermilk, yogurt, sour milk, kefir, fruit yogurt, cream, crème fraîche, cream cheese, cottage cheese, soft and hard cheese, acid curd cheese, desserts
Ratio unsaturated:saturated fatty acids[1]	Polyunsaturated fatty acids, monounsaturated fatty acids, saturated fatty acids
Alcohol	Beer, Wheat beer, stout, wine (white, rosé, red, sparkling), liqueurs, spirits, mixed alcoholic drinks
**Healthy Nordic Food Index**	
Apples and pears[1]	Apple, pear
Cabbage[1]	Sauerkraut, broccoli, cauliflower, white and red cabbage, kale, brussel sprouts
Root vegetables[1]	Carrot
Fish[1]	Fish, ready-to-eat fish dishes
Oats and cereals[1]	Oats, other breakfast cereals
Wholegrain bread[1]	Wholegrain bread and bread rolls

[1] *Food groups scored positively for calculation of dietary pattern score.*
[2] *Food groups scored negatively for calculation of dietary pattern score.*

Online Resource 3: Characterization of median daily macronutrient intake and intake of food groups of the study sample used to calculate dietary pattern scores stratified by mortality status at mortality follow-up.

	All (n=836)	Deceased[5] (n=93)	Alive[5] (n=743)
**Macronutrients [g/d][4]**			
**Protein**	77.0 [70.4; 84.2]	77.8 [70.8; 88.7]	76.8 [70.3; 83.8]
**Carbohydrates**	220.4 [202.6; 238.5]	222.4 [197.6; 238.8]	220.4 [203.2; 238.5]
**Fat**	97.8 [91.3; 105.1]	98.7 [90.5; 107.9]	97.8 [91.4; 104.5]
**Fiber**	21.4 [19.0; 24.5]	21.9 [18.5; 24.9]	21.4 [19.1; 24.4]
**Alcohol[2]**	10.5 [5.5; 18.5]	10.7 [3.6; 16.2]	10.5 [5.7; 18.7]
**Food groups [g/d][4] or [mL/d][4]**			
**Fruits[1]**	184.0 [130.8; 292.3]	191.1 [131.9; 315.9]	183.9 [130.7; 291.2]
**Fruits and nuts[2]**	186.6 [132.8; 300.5]	193.9 [141.1; 318.1]	185.4 [132.2; 297.1]
**Apples and pears[3]**	74.9 [31.5; 224.1]	76.6 [32.0; 224.1]	74.8 [31.5; 224.1]
**Vegetables[1, 2]**	180.5 [146.8; 218.0]	174.5 [153.3; 212.3]	181.4 [145.8; 218.1]
**Root vegetables[3]**	18.4 [14.6; 28.5]	17.3 [12.1; 12.1]	18.5 [14.8; 28.9]
**Cabbage[3]**	23.3 [18.1; 30.3]	23.4 [18.0; 31.4]	23.3 [18.1; 30.0]
**Legumes[2]**	1.9 [1.2; 4.1]	2.2 [1.5; 6.1]	1.9 [1.2; 3.9]
**Nuts and legumes[1]**	6.7 [4.6; 10.6]	7.3 [4.3; 10.6]	6.7 [4.6; 10.7]
**Cereals[2]**	149.0 [115.4; 181.9]	143.7 [109.1; 176.6]	149.5 [116.0; 183.2]
**Whole grains[1]**	67.5 [48.9; 85.0]	64.5 [51.4; 86.1]	68 [48.8; 84.9]
**Wholegrain bread[3]**	30.8 [18.9; 44.4]	29.4 [18.4; 43.6]	31.1 [19.0; 44.4]
**Oats and cereals[3]**	0.9 [0.2; 6.6]	0.4 [0.0; 2.5]	1.1 [0.3; 6.6]
**Dairy products[2]**	228.4 [154.7; 336.0]	246.9 [164.2; 358.1]	225.8 [154.3; 333.0]
**Low-fat dairy products[1]**	14.6 [0.7; 68.3]	13.3 [0.7; 55.0]	15.3 [0.7; 72.2]
**Meat and poultry[2]**	109.2 [80.6; 140.4]	113.6 [84.1; 161.6]	108.5 [79.3; 139.4]
**Red and processed meat[1]**	45.3 [27.3; 66.3]	53.4 [30.8; 77.7]	44.6 [27.0; 65.1]
**Fish[3]**	23.0 [11.7; 37.6]	20.1 [9.1; 34.8]	23.1 [12.0; 37.7]
**Fish and seafood[2]**	23.7 [12.4; 38.3]	20.8 [9.8; 35.5]	23.8 [12.7; 38.4]
**Sweetened beverages[1]**	70.8 [-20.4; 153.9][6]	58.1 [-53.6; 152.5][6]	73.7 [-17; 153.9][6]
**Ratio unsaturated: saturated fatty acids[2]**	1.3 [1.2; 1.5]	1.3 [1.1; 1.5]	1.3 [1.2; 1.5]
**Dietary sodium[1]**	2.3 [2.1; 2.5]	2.4 [2; 2.6]	2.3 [2.1; 2.5]

[1] Food group included in Dietary Approaches to Stop Hypertension score.
[2] Food group included in Modified Mediterranean Diet Score.
[3] Food group included in Healthy Nordic Food Index.
[4] Intake adjusted for total energy intake using residual method and subsequent addition of a constant based on mean energy intake of the study population. Dietary assessment was undertaken at the second examination cycle of the popgen control cohort.
[5] At mortality follow-up in 2022.
[6] Negative values due to high standard deviation caused by an intake of >1.8L soft drinks/d in ~1% of the study population.

Online Resource 4: Association of energy-adjusted food groups comprising the Healthy Nordic Food Index with all-cause mortality using Cox proportional hazard regression models.

	**Low intake (< Median)[1]**	**High intake (≥ Median)**
**Cabbage**		
Individuals, n (%)	418 (50.0%)	418 (50.0%)
Deceased, n (%)	48 (11.4%)	45 (10.8%)
Model 1	Ref.	0.83 [0.55–1.24]
Model 2	Ref.	0.89 [0.59–1.35]
**Root vegetables**		
Individuals, n (%)	418 (50.0%)	418 (50.0%)
Deceased, n (%)	51 (12.2%)	42 (10.1%)
Model 1	Ref.	0.79 [0.53–1.19]
Model 2	Ref.	0.81 [0.54–1.23]
**Apples and pears**		
Individuals, n (%)	418 (50.0%)	418 (50.0%)
Deceased, n (%)	46 (11.0%)	47 (11.2%)
Model 1	Ref.	0.76 [0.50–1.15]
Model 2	Ref.	0.80 [0.52–1.23]
**Fish**		
Individuals, n (%)	418 (50.0%)	418 (50.0%)
Deceased, n (%)	53 (12.7%)	40 (9.6%)
Model 1	Ref.	0.67 [0.44–1.00]
Model 2	Ref.	0.69 [0.45–1.04]
**Oats and cereals**		
Individuals, n (%)	418 (50.0%)	418 (50.0%)
Deceased, n (%)	61 (14.6%)	32 (7.7%)
Model 1	Ref.	**0.55 [0.36–0.85]**
Model 2	Ref.	**0.59 [0.38–0.91]**
**Wholegrain bread**		
Individuals, n (%)	418 (50.0%)	418 (50.0%)
Deceased, n (%)	50 (12.0%)	43 (10.3%)
Model 1	Ref.	0.92 [0.61–1.39]
Model 2	Ref.	0.92 [0.61–1.40]

[1] Low intake group (intake < sample median) was set as reference.
For survival analyses, intake of food groups was adjusted for energy intake by residual method and subsequent addition of a constant based on mean energy intake of the study population.
Associations are in Hazard Ratios [95% Confidence Interval]. Bold values indicate significant associations.
Model 1: adjusted for sex and age.
Model 2: Model 1 further adjusted for Body Mass Index, Waist to Hip Ratio, education, smoking status, total energy intake, and physical activity.

Online Resource 5: Association of variations of the energy-adjusted HNFI with all-cause mortality using Cox proportional hazard regression models after separate exclusion of each single food group from the full score.

	**1-SD increment**
**Full HNFI**	
Model 1	0.76 [0.62–0.94]
Model 2	0.79 [0.64–0.98]
**HNFI minus cabbage**	
Model 1	0.77 [0.62–0.95]
Model 2	0.80 [0.64–1.00]
**HNFI minus root vegetables**	
Model 1	0.79 [0.64–0.97]
Model 2	**0.88 [0.71–1.10]**
**HNFI minus apples and pears**	
Model 1	0.75 [0.75–0.92]
Model 2	0.77 [0.62–0.96]
**HNFI minus fish**	
Model 1	0.80 [0.80–0.98]
Model 2	**0.85 [0.69–1.06]**
**HNFI minus oats and cereals**	
Model 1	0.80 [0.80–0.99]
Model 2	**0.86 [0.69–1.07]**
**HNFI minus wholegrain bread**	
Model 1	0.74 [0.74–0.91]
Model 2	0.76 [0.61–0.94]

Model 1: adjusted for sex and age.
Model 2: Model 1 further adjusted for Body Mass Index, Waist to Hip Ratio, education, smoking status, total energy intake, physical activity, and respective excluded food group.
Bold Hazard Ratios [95% Confidence Interval] indicate an association of modified HNFI with mortality risk that fails to reach statistical significance (p>0.05).
HNFI, Healthy Nordic Food Index; SD, standard deviation.
